# Pan-genomic insights into resistance, virulence, and stress adaptation in *Clostridium perfringens* from the Tibetan Plateau

**DOI:** 10.1128/msphere.00792-25

**Published:** 2026-04-20

**Authors:** Runbo Luo, Yanxi Wan, Wanting Chen, Hongyun Wei, Junyang Wang, Zhanchun Bai, Kexin Li, Yanan Zhong, Bin Ma, Hui Jin, Sizhu Suolang

**Affiliations:** 1 Key Laboratory for Prevention and Control of Hydatid Disease in Xizang (Co-constructed by Ministry and Province), Ministry of Agriculture and Rural Affairs, College of Animal Science, Xizang Agricultural and Animal Husbandry University, Linzhi, Xizang, China; 2 State Key Laboratory of Agricultural Microbiology, College of Veterinary Medicine, Huazhong Agricultural University, Wuhan, China; 3 Key Laboratory of Preventive Veterinary Medicine in Hubei Province, The Cooperative Innovation Center for Sustainable Pig Production, Wuhan, China; University of Wisconsin-Madison, Madison, Wisconsin, USA

**Keywords:** *Clostridium perfringens*, Bacteriology of the Qinghai-Tibet Plateau, Veterinary Microbiology, genomics, molecular epidemiology

## Abstract

*Clostridium perfringens* is a ubiquitous foodborne and zoonotic pathogen responsible for necrotizing enteritis, food poisoning, and gas gangrene in humans and livestock, with mortality reaching 100% in specific subtypes. The Qinghai-Tibetan Plateau, with its high altitude, hypoxia, large temperature fluctuations, and strong UV radiation, imposes a strong selective regime that drives distinctive, plateau-specific microbial evolution. We isolated and sequenced eight *C. perfringens* isolates from yaks and Tibetan pigs and analyzed them with 129 public genomes. Multilocus sequence typing (MLST) revealed extensive genetic heterogeneity, with 35 novel alleles and 18 novel sequence types (STs), all plateau isolates carrying novel alleles or STs. Toxinotyping identified six toxinotypes, dominated by A (43.0%) and F (42.3%), with virulence gene repertoires matching toxinotype assignments. Sixteen antimicrobial resistance (AMR) genes across eight drug classes were detected, with vancomycin- and defensin-like genes present in >96.0% of isolates; four were linked to mobile genetic elements (MGEs) or type IV secretion systems (T4SS). Environmental tolerance assays showed that three plateau isolates maintained growth at pH 7.5 under anaerobic conditions, while the growth of all isolates was inhibited at pH 7.0. Pan-genomic association analysis identified a 146-node, 3,390-edge network, with *IS* family transposases (*ISCbt3/ISCpe4*), sigma factors (*sigL* and *sigA*), and DNA polymerase I (*polA*) as central hubs potentially mediating stress adaptation. Genomic epidemiology of *C. perfringens* from the Tibetan Plateau reveals marked genetic diversity, broad resistance, toxinotype-defined virulence, and genetic signatures of high-altitude adaptation, underpinning evidence-based control in plateau ecosystems.

**IMPORTANCE:**

*Clostridium perfringens* is a widespread pathogen, but its adaptation to extreme environments like high-altitude plateaus remains a mystery. Our integrated genomic analysis of 137 strains, including 8 newly sequenced from the plateau, uncovers a startling reality: this region harbors a highly diverse population with near-universal resistance to critical antibiotics. We identified new sequence types (STs) and genetic “hubs” that may drive this adaptation. These findings have profound implications for One Health as they highlight an environmental niche where resistance can evolve and potentially spread, underscoring the urgent need for surveillance in unique ecosystems.

## INTRODUCTION

*Clostridium perfringens* is a gram-positive anaerobe that holds considerable clinical and epidemiological importance in both human medicine and veterinary sciences. Widely distributed in soil, water, food, and the gastrointestinal tracts of humans and animals, it is a leading cause of necrotizing enteritis, food poisoning, gas gangrene, and other toxin-mediated diseases across a broad host range (1–6). Its pathogenicity is driven by the rapid proliferation of vegetative cells and the production of diverse protein toxins, including the four principal typing toxins (α, β, ε, and ι) and multiple accessory virulence factors. Based on the presence and combination of major toxins, *C. perfringens* strains are classified into seven toxinotypes (A–G), each of which is associated with characteristic disease manifestations in humans and animals (7–9). In humans, type F strains producing enterotoxin (*cpe*) are a major cause of foodborne diarrhea worldwide, accounting for millions of cases annually, while type C strains can induce fatal necrotizing enteritis, historically known as “pig-bel” in Papua New Guinea and parts of Asia (10–15). In livestock, type A and G strains cause necrotic enteritis in poultry, leading to substantial mortality and reduced productivity; type C strains cause hemorrhagic enteritis in piglets, with case fatality rates approaching 100% (16–18); and type D strains are responsible for enterotoxemia in sheep and goats, often leading to sudden death and significant economic losses (10–12, 19–25).

The Qinghai-Tibetan Plateau, the highest and one of the most extreme inhabited regions on Earth, is characterized by hypobaric hypoxia, strong ultraviolet radiation, and large diurnal temperature fluctuations. These environmental pressures can act as potent selective forces shaping microbial genomes, potentially driving unique adaptations in virulence, resistance, and stress tolerance (26–30). A study of *C. perfringens* from yaks on the Qinghai-Tibet Plateau reported an extremely high rate of multidrug resistance (98.6% resistant to ≥4 antibiotics) and pronounced genetic diversity, identifying 89 sequence types (STs) among 144 isolates using PCR typing (31). Moreover, an isolate from Tibetan sheep (QHY-2) harbored a novel *optrA*-positive plasmid (pQHY-2) carrying a *Tn6218*-like transposon that can mediate resistance to last-resort antibiotics such as tigecycline and co-select for multiple resistance determinants (e.g., *fexA* and *erm*) (32). These findings imply potential horizontal transfer of resistance determinants among plateau isolates and suggest that plateau *C. perfringens* populations may serve as important reservoirs of antimicrobial resistance (AMR) genes. However, these investigations were largely based on phenotypic analyses or limited genotyping and have not systematically elucidated the mechanisms and evolutionary dynamics of resistance transmission at the whole-genome level. Despite its ecological significance, *C. perfringens* from this region remains underexplored, and no comprehensive genomic-epidemiological study based on whole-genome sequencing (WGS) has been reported.

Although *C. perfringens* has traditionally been considered susceptible to many antimicrobials, extensive antibiotic use in veterinary medicine and livestock production has likely contributed to the global emergence of multidrug-resistant strains (33, 34). While most surveillance efforts have focused on isolates from intensive farming systems or clinical outbreaks, the prevalence and genetic basis of antimicrobial resistance in *C. perfringens* from plateau regions remain largely unexplored (35). The Qinghai-Tibetan Plateau, with its unique ecological conditions and distinctive patterns of antimicrobial usage, provides a critical yet understudied setting to investigate how environmental pressures and local husbandry practices shape the resistance gene repertoire of this pathogen.

In this study, we performed WGS and comparative genomic analyses of eight *C. perfringens* isolates from yaks and Tibetan pigs on the Qinghai-Tibetan Plateau, integrating these with 129 publicly available genomes. We systematically characterized multilocus sequence typing (MLST) diversity, toxinotypes, virulence-associated factors, and AMR gene repertoires. Environmental tolerance assays were conducted to assess growth under different pH conditions, and gene co-occurrence networks were constructed to identify candidate determinants of stress adaptation. This study comprehensively integrated molecular epidemiology and comparative genomics methods, providing a solid foundation for understanding the ecological adaptation and pathogenic mechanism of *C. perfringens* in Tibetan areas.

## MATERIALS AND METHODS

### Bacterial isolation and culture conditions

A total of eight *C. perfringens* strains were collected from the Qinghai-Tibetan Plateau since 2019. Details of the sources and characteristics of these strains are provided in Table 1. Primary colonies were anaerobically inoculated onto TSC medium and incubated at 43°C for 24 h. Thereafter, black-pigmented single colonies were aseptically picked and transferred into 5 mL of RCM medium under strict anaerobic conditions to propagate the F₂ generation for subsequent collection.

**TABLE 1:** Basic information of eight *Clostridium perfringens* isolates

Strain no.	Host	Isolation source	Collection site
A37	Tibetan pig	Feces	Xizang Autonomous Region, China
A44	Tibetan pig	Feces	Xizang Autonomous Region, China
ZD_8	Yak	Feces	Qinghai Province, China
ZD_9	Yak	Feces	Qinghai Province, China
BG_3	Yak	Feces	Xizang Autonomous Region, China
DT_10	Yak	Feces	Qinghai Province, China
DT_17	Yak	Feces	Qinghai Province, China
LZ_1	Yak	Feces	Xizang Autonomous Region, China

Furthermore, 129 *C. perfringens* genomes were retrieved from the National Center for Biotechnology Information (NCBI) database (accessed on 20 May 2025; https://www.ncbi.nlm.nih.gov/), with metadata on host, sample type, and geographic location extracted from GenBank annotations. Among the publicly available *C. perfringens* genomes in GenBank, isolates originating from South Korea were most frequently represented (*n* = 45), followed by those from China (*n* = 30). Strains from Finland, Germany, the United States, Ghana, and Japan constituted intermediate proportions, whereas Canada, Australia, and Norway were represented by only a single isolate each.

To provide a comprehensive context for the comparative analysis with the Tibetan Plateau isolates, we systematically categorized the 129 public genomes by host source. Host categories were assigned based on the GenBank “host” field or inferred from the “isolation_source” when unambiguous (e.g., chicken and bovine). Detailed metadata for all public genomes, including accession numbers, host categories, isolation sources, and geographic origins, are provided in Table S1.

With respect to sample sources, fecal specimens were predominant (*n* = 37), consistent with the well-recognized gastrointestinal niche of *C. perfringens*. Aquatic and sludge samples (*n* = 10), food and food-associated samples (*n* = 9), intestinal contents (*n* = 6), human clinical samples (*n* = 4), animal host samples (*n* = 3), and environmental sources (*n* = 2) were comparatively less common. Regarding the host distribution, human-derived isolates accounted for the largest proportion (*n* = 28), followed by livestock and poultry (*n* = 27) and companion animals (*n* = 12); 11 isolates could not be assigned to a specific host category.

### Genomic DNA extraction and library preparation

Genomic DNA was extracted from *C. perfringens* cells using the E.Z.N.A. Tissue DNA Kit (Omega Bio-Tek) according to the manufacturer’s Tissue DNA-Spin Protocol. DNA quality was assessed after extraction, and only samples with a total yield > 3 µg, concentration > 30 ng/µL, and OD260/OD280 between 1.80 and 2.00 were used for library preparation. For each isolate, 3 µg of high-quality genomic DNA was utilized to construct paired-end sequencing libraries (insert size ~450 bp) in accordance with standard MGI-T7 protocols (Shanghai BIOZERON Biotech Co., Ltd., Shanghai, China). Genomic DNA was fragmented into the desired size range using a Covaris ultrasonicator. The fragments were then end-repaired with T4 DNA polymerase and adenylated at the 3′ termini. Sequencing adapters were ligated to the A-tailed fragments, which were subsequently subjected to size selection by agarose gel electrophoresis. The fragments were then enriched by PCR, with index tags incorporated during amplification. Finally, the libraries underwent quality control and were sequenced on the MGI-T7 platform in the PE150 mode according to the manufacturer’s instructions.

### Genome assembly and average nucleotide identity (ANI) analysis

Raw FASTQ reads were *de novo-*assembled into contiguous sequences using SPAdes v4.0.0, resulting in FASTA-formatted genome assemblies (36). A total of 129 *C. perfringens* whole-genome sequences were retrieved from the NCBI database as reference genomes. These, together with the eight newly sequenced isolates, comprised a total of 137 genomes subjected to ANI analysis using FastANI v1.34 (37).

### Pan-genome construction and phylogenomic analysis

A total of 137 *C. perfringens* genomes were incorporated into a unified pan-genome and phylogenomic analysis pipeline. Annotation was performed with Prokka v. 1.14.6, followed by pan-genome reconstruction using Panaroo v. 1.5.0. Core-gene alignments produced by Panaroo v. 1.5.0 (.aln files) were then subjected to maximum-likelihood phylogenetic inference in IQ-TREE v. 2.4.0. The resulting phylogeny was visualized in R v. 4.4.0 with ggplot2 v. 3.5.1 and subsequently polished in Adobe Illustrator 2024 (38–41). Each sequence was manually annotated based on the phylogenetic context and prior literature. Finally, the phylogenetic tree was visualized by TVBOT and retouched using Adobe Illustrator 2024 (42).

### MLST and virulence typing

Sequence types for each isolate were determined using FastMLST v0.0.16 with default parameters based on the *C. perfringens* typing scheme from the PubMLST database (43). Furthermore, comparative genomic analyses for molecular virulence typing were conducted by querying each *C. perfringens* genome against a species-specific virulence gene nucleotide database using TOXIper v1.1 under default settings (44).

### Identification of virulence factors and antimicrobial resistance genes

All *C. perfringens* genome sequences were searched against the Virulence Factor Database (VFDB) using the BLASTn algorithm. The BLASTn minimum identity was set to 90.0%, and the minimum coverage was set to 80.0% (45). The antibiotic resistance genes (ARGs) of each strain were identified by RGI v. 6.0.3 using reference data from the Comprehensive Antibiotic Resistance Database (CARD) (46). Python 3.12.13 scripts were then used to collect matching gene fragments for downstream analysis and visualization.

### Annotation of mobile genetic elements (MGEs)

Gene fragments identified as virulence factors (VFs) or ARGs in each strain were further annotated using the ICEberg 3.0 database to classify them as integrative and conjugative elements (ICEs), type IV secretion systems (T4SSs), integrative mobilizable elements (IMEs), or conjugative insertional mobile elements (CIMEs) (47). BLASTn was used to align each gene fragment against the ICEberg 3.0 database, with a minimum identity of 90.0% and minimum coverage of 80.0%. Matched gene fragments were extracted using custom Python scripts. Furthermore, the data sets comprising VFs, ARGs, and ICEs from 137 *C. perfringens* genomes were visualized using the ggplot2 v. 3.5.1 and ComplexHeatmap v. 2.20.0 in R 4.4.0, with subsequent editing conducted in Adobe Illustrator 2024 (41, 48).

### Acid-base tolerance assays

In the acid-base environment tolerance assay, eight isolates were obtained from single colonies grown on double-layer TSC plates and cultured overnight in RCM medium. The cultures were then diluted 1:100 and incubated for 5 h. Subsequently, 5 µL of the culture was added to 120 µL of RCM medium in 96-well plates, resulting in a total volume of 125 µL per well. The samples were incubated at three pH levels (7.0, 7.5, and 8.0) under anaerobic conditions at 45°C.

This pH range was selected to represent a near-neutral to mildly alkaline range that is relevant to gastrointestinal environments and potential pH variation associated with yak and Tibetan pig hosts and plateau-related conditions (49). To ensure experimental robustness, three independent biological replicates were performed for each strain under each condition.

Optical density at 630 nm (OD₆₃₀) was recorded every 30 min using an automated microbial growth curve analyzer (MGC-500, Ningbo Scientz Biotechnology Co., Ltd., Ningbo, China), and the growth curve was plotted after the measurement was completed. Since pH 8.0 represented the optimal growth condition in this study, it was selected as the reference for statistical comparisons. Differences in growth between this optimum and the other pH levels (7.0 and 7.5) were analyzed using two-tailed paired *t*-tests (*P* < 0.05). Finally, potential genomic correlates of tolerance were evaluated by comparing quantitative gene counts (ARGs and MGEs) via unpaired *t*-tests and categorical toxinotype distributions via Fisher’s exact test.

### Gene co-occurrence network analysis

Based on the environmental tolerance assay results, the eight isolates were classified into tolerant (*n* = 3) and nontolerant (*n* = 5) groups. A correlation-based comparative analysis was performed using the gene presence-absence matrix generated by Panaroo v1.5.0 and implemented in PPNet, with a correlation coefficient threshold of ≥0.5 and a *P*-value threshold of ≤0.5 to identify significant gene-gene associations (50).

In this network model, “nodes” represent orthologous gene clusters identified by Panaroo, representing functional genetic determinants. “Edges” represent pairwise correlations satisfying the aforementioned thresholds; biologically, an edge implies a “co-occurrence” relationship, suggesting that connected genes are likely co-selected or functionally linked to facilitate adaptation to the plateau environment (51, 52). The resulting gene association network was visualized using Cytoscape v3.10.3, applying a concentric circle layout algorithm that partitioned all gene nodes into degree-based intervals and mapped them hierarchically onto circles with varying radii.

## RESULTS

### Comparative genomics demonstrates dispersed clustering of plateau-derived isolates

A total of 137 *C. perfringens* genomes were analyzed in this study, comprising 8 newly isolated strains from fecal samples of yaks (*n* = 6) and Tibetan pigs (*n* = 2) collected on the Tibetan Plateau, together with 129 publicly available genomes retrieved from the NCBI database (https://www.ncbi.nlm.nih.gov/; strain accession numbers and other raw data are listed in Table S1).

**Fig 1:**
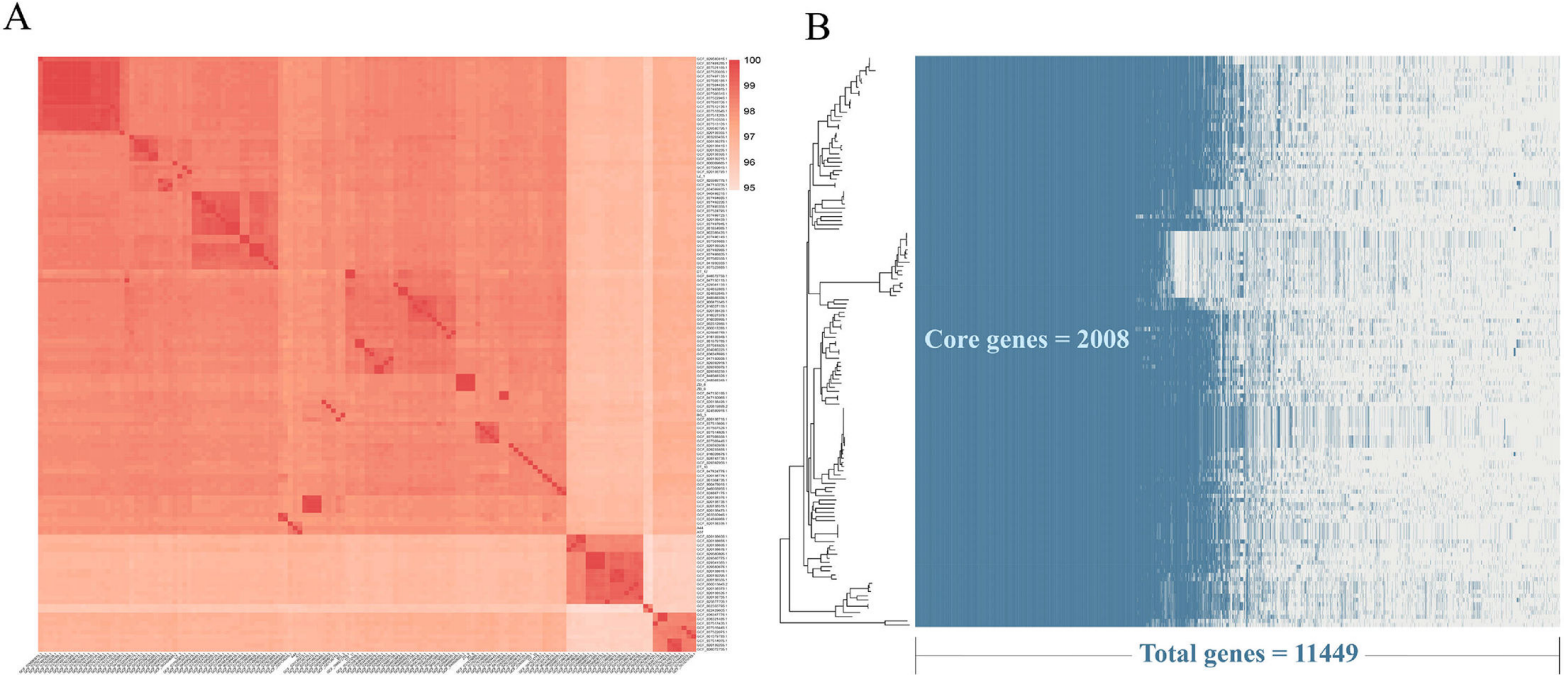
(**A**) The heatmap displays pairwise ANI values calculated using FastANI for all 137 *Clostridium perfringens* genomes. Rows and columns correspond to individual strain isolates and are arranged according to hierarchical clustering. The color gradient from 95.0% to 100.0% denotes pairwise ANI values, with deeper red hues indicating greater genomic similarity. (**B**) The figure presents a pan-genome presence-absence heatmap for 137 *C. perfringens* strains alongside a phylogenetic tree inferred from core genes. The pan-genome was constructed using Panaroo, and core genes were defined as those present in ≥99% of strains (*n* = 2,008). The left panel depicts the phylogenetic tree derived from core-genome alignments and inferred using IQ-TREE with 1,000 Ultrafast Bootstrap (UFBoot) replicates, whereas the right panel presents a heatmap in which gene presence is indicated in blue and absence in white. Annotations specify the total pan-genome gene count (*n* = 11,449), highlighting conserved (core) versus strain-specific (non-core) gene distribution patterns.

As summarized in Table 2, human-derived isolates constituted the largest specified group (*n* = 31, 24.0%), followed by livestock and poultry (*n* = 27, 20.9%), companion animals (*n* = 12, 9.3%), and other animals (*n* = 8, 6.2%). Despite these efforts, a significant proportion of the public data sets (*n* = 51, 39.5%) lacked specific annotations and were therefore classified as “Unknown.” ANI analysis of the 137 *C. perfringens* genomes revealed pairwise ANI values exceeding 95.0% (Fig. 1A). These results confirmed that all isolates belonged to *C. perfringens* (53).

**TABLE 2:** Distribution of host sources among the 129 publicly available *Clostridium perfringens* genomes

Host category	*n*	%
Human	31	24
Livestock/poultry	27	20.9
Companion animals	12	9.3
Other animals	8	6.2
Unknown	51	39.5
Total (public genomes only)	129	100

The genomes of these strains were annotated, and the pan-genome was subsequently constructed. A total of 2,008 core genes and 11,449 pan-genome genes were identified. Finally, a phylogenetic tree was reconstructed based on single-nucleotide polymorphisms (SNPs) within the core gene set (Fig. 1B). It revealed clear separation of strains into multiple clades, largely consistent with their toxinotypes and sequence types. Notably, plateau isolates were distributed across distinct branches rather than forming a single cluster, suggesting multiple independent lineages rather than a geographically restricted genotype.

**Fig 2:**
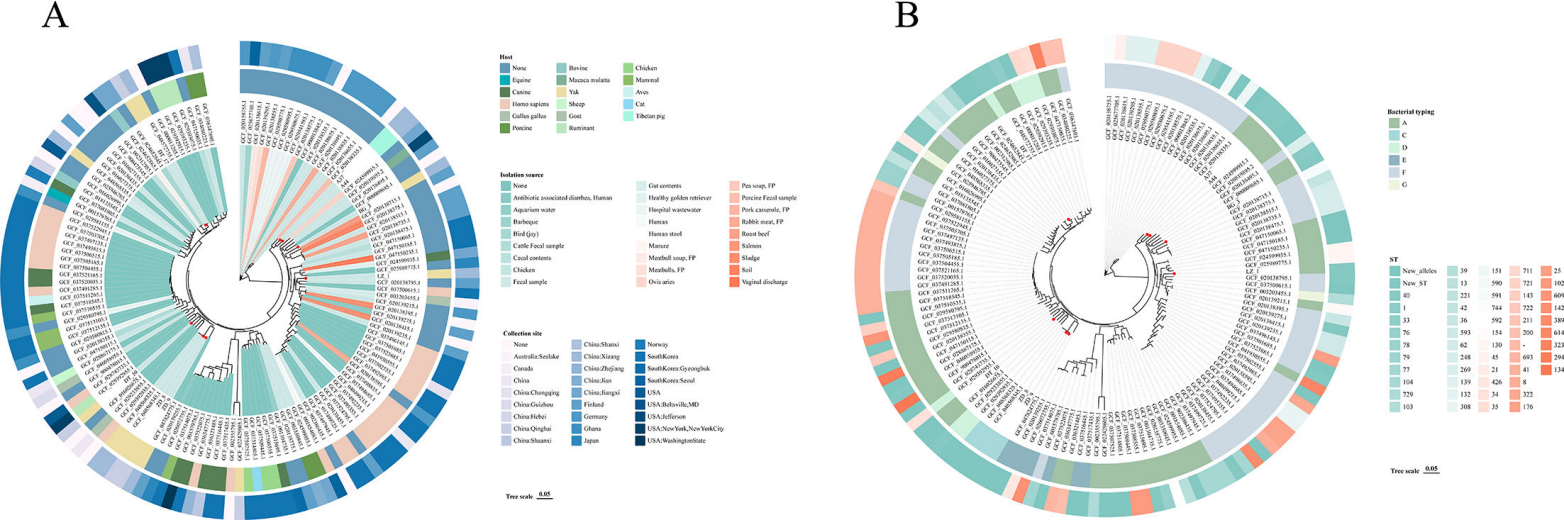
(**A**) The circular phylogenetic tree illustrates the evolutionary relationships among 137 *Clostridium perfringens* strains based on their core-genome SNPs, derived from the Panaroo-defined core genome. Evolutionary lineages are color-coded by strain metadata, and the outer ring is annotated from outside inward with the geographic collection site, sample source, and host origin, each category being distinguished by its own hue. On the phylogenetic tree, strains are marked with “•.” The legend indicates a tree scale of 0.05, representing genetic distance. (**B**) Molecular typing annotations for each strain are displayed on the phylogenetic tree; the inner ring indicates bacterial toxinotype, while the outer ring indicates the ST.

Notably, the Tibetan Plateau isolates of toxinotype C formed a distinct monophyletic lineage. In particular, ZD_8 and ZD_9 (this study) clustered with two public genomes (GCF_048568345.1 and GCF_048568325.1) to form a four-member clade. Examination of the associated metadata showed strong concordance, with all isolates in this clade originating from Tibet and classified as toxinotype C. Importantly, they were the only four toxinotype C isolates identified across both our data set and the referenced genomes (Fig. 2B). This phylogenetic clustering therefore suggests the presence of a geographically restricted lineage potentially linked to a specific toxin profile.

### MLST analysis of 137 *C. perfringens* genomes

To assess population structure and lineage diversity, MLST analysis was performed on 137 *C. perfringens* genomes, comprising 8 newly sequenced isolates in this study and 129 publicly available GenBank assemblies, which revealed 55 confirmed STs, 30 novel allelic combinations, and 17 novel STs.

Host metadata indicated that human-derived isolates were the largest single category (*n* = 28), whereas sample-type information showed a predominance of fecal specimens (*n* = 34) (Fig. 2A). In the MLST analysis, 35 strains across the entire genome were found to carry the newly identified alleles, and 18 strains represented novel STs. Among previously described STs, ST41 was the most frequent (*n* = 12, 8.8%), followed by ST721 and ST77 (*n* = 4 each, 2.9%) and ST248 and ST139 (*n* = 3 each, 2.2%); all remaining STs occurred at frequencies ≤2 isolates. Toxinotypes A and F displayed the greatest genotypic diversity, each encompassing 28 distinct STs, whereas all type C isolates (*n* = 4) exhibited allele combinations not previously recorded in public MLST databases. Notably, all eight plateau isolates carried novel alleles and/or were assigned to novel STs, a pattern that may indicate regionally distinct genotypes pending confirmation by broader sampling (Fig. 2B). Phylogenetic reconstruction corroborated the extensive diversity of both toxinotypes and STs across the data set, consistent with a heterogeneous population structure.

### Toxinotyping and association with virulence factors of *C. perfringens* genomes

We employed TOXIper v1.1 for toxinotyping and systematically identified virulence-associated genes across the same set of 137 genomes. Toxinotyping identified six toxinotypes: type A was most prevalent (*n* = 59, 43.1%), followed closely by type F (*n* = 58, 42.3%); types E and D were each represented by seven strains, type C by four strains, and type G by two strains (Fig. 2B). Among the eight isolates obtained from the Qinghai-Tibet Plateau, five were assigned to toxinotype A (A37, A44, BG_3, DT_10, and LZ_1), two to toxinotype C (ZD_8 and ZD_9), and one to toxinotype D (DT_17). Collectively, toxinotypes A and F accounted for 117 of 137 genomes (85.4%), indicating a marked predominance of these two toxin profiles within the current data set.

**Fig 3:**
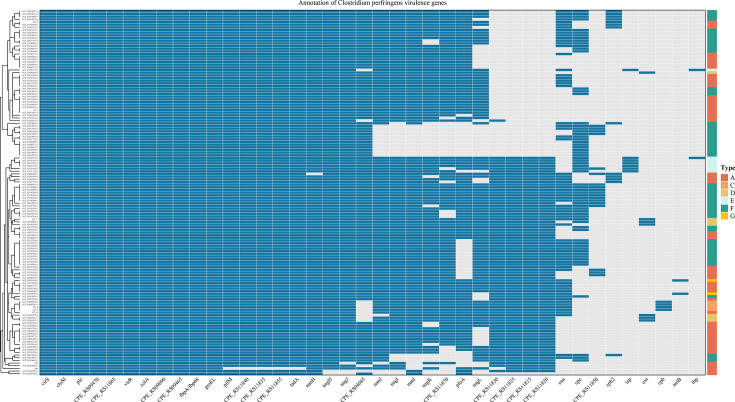
The presence-absence heatmap illustrates the correlation between virulence gene repertoires and toxinotypes among *Clostridium perfringens* strains (*n* = 137). In the heatmap, gene presence is indicated by blue squares and absence by gray. Strains are hierarchically clustered on the left based on their virulence gene profiles, revealing the heterogeneity within the population. The x-axis lists the principal virulence-associated genes (identity ≥ 90.0%; coverage ≥ 80.0%), including major toxins and other determinants, while the legend on the right categorizes each strain by its toxin type (A–G), denoted by a unique color.

To link population structure with virulence potential, we systematically annotated virulence-associated loci across 137 genomes and mapped their distribution against established toxinotypes (Fig. 3). Across the data set, we identified 11 widely distributed virulence determinants that together constitute a conserved core virulome within the collection. These include the two-component regulatory system *virS/virR*, the collagenase gene *colA*, phospholipase C (*plc/cpa*), the fibronectin-binding protein *fbpA*, the molecular chaperone *groEL*, and four members of the *CPE_RS* family. Classical typing markers exhibited toxinotype-restricted distributions: the β-toxin gene *cpb* was confined to toxinotypes B and C, the ε-toxin gene *etx* to toxinotypes B and D, and the iota locus (*iap*/*ibp*) uniquely characterized type E isolates. More recently described virulence factors demonstrated toxinotype-associated patterns: *cpe* predominated in type F but was occasionally observed in types C–E, *netB* was effectively confined to type G (consistent with its role in avian necrotic enteritis), and *cpb2*, while most frequently detected in type A, was found across multiple toxinotypes. Distinct toxinotypes, therefore, displayed characteristic virulence-gene complements, and the pronounced MLST diversity observed in types A and F paralleled increased heterogeneity in accessory virulence-gene carriage.

Genome annotation of the eight plateau isolates demonstrated that *plc* (α-toxin/*cpa*) was highly conserved across these local genomes. By contrast, canonical variable loci (*cpb*, *cpb2,* and *cpe*) exhibited a sporadic distribution among the plateau isolates, indicating heterogeneous pathogenic potential within this local collection rather than a uniform virulence profile. Notably, the yak-derived isolate BG_3 was found to carry the β2-toxin gene (*cpb2*), indicating presence of this accessory toxin in at least one local strain. All plateau isolates commonly harbored genes implicated in adhesion and surface colonization (for example, *fbpA*, *pilM,* and *tadA*), a pattern consistent with an intestinal colonization phenotype (54–57). Together, these results, which combine broad-scale toxinotyping and virulence profiling across 137 genomes with focused genomic characterization of plateau isolates, highlight the value of WGS as a tool for integrated toxinotyping, virulence profiling, and molecular surveillance of *C. perfringens*.

### Antibiotic resistance phenotypes and genes

**Fig 4:**
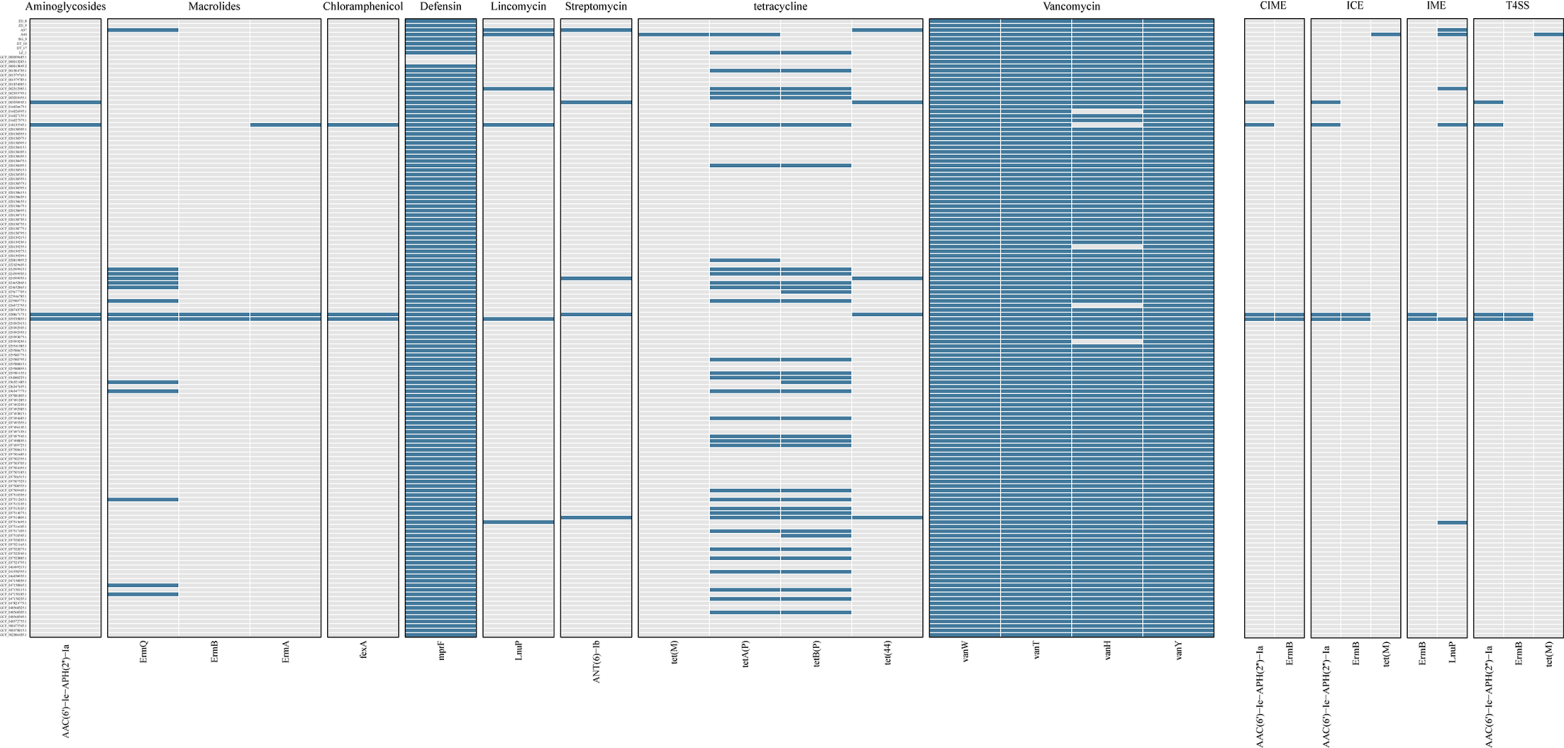
This presence-absence heatmap reveals the distribution and diversity of antibiotic-resistance determinants and MGE-associated genes across 137 *Clostridium perfringens* isolates. In the map, rows represent individual isolates (*n* = 137), while columns are grouped into 12 functional categories: eight antibiotic resistance gene classes and four MGE classes (CIME, ICE, IME, and T4SS) identified using RGI v6.0.3 and ICEberg 3.0 (identity ≥ 90.0%; coverage ≥ 80.0%). Gene presence is indicated by blue squares and absence by light-gray squares. The pattern of gene distribution highlights the prevalence of specific resistance determinants and underscores their potential for horizontal transfer via MGEs, providing insights into the evolutionary dynamics of resistance in this species.

To elucidate the genomic determinants underlying antibiotic resistance in 137 *C. perfringens* isolates, we conducted a systematic identification of ARGs within their whole-genome sequences. Our analysis revealed enrichment of ARGs spanning eight antibiotic classes, with sixteen unique ARGs detected. Notably, four ARGs were associated with MGEs and type IV secretion systems. Among the three MGE categories (CIMEs, ICEs, and IMEs), the bifunctional aminoglycoside acetyltransferase/phosphotransferase *AAC(6*′*)-Ie-APH(2*″*)-Ia* and the macrolide methyltransferase *ErmB* exhibited the highest prevalence (Fig. 4).

Vancomycin-related and tetracycline-related determinants were the most abundant ARG classes, each represented by four genes [*vanW*, *vanT*, *vanH*, and *vanY; tetB(P)*, *tet(M*), *tet(44)*, and *tetA(P)*]. Macrolide-resistance determinants (*ermB*, *ermQ*, and *ermA*) were the next most prevalent ARGs. All 137 genomes were found to harbor three vancomycin resistance genes (*vanW*, *vanT*, *and vanY*). Both vancomycin and defensin classes of resistance genes were detected in over 96.0% of the genomes. In contrast, all other ARG classes were found in fewer than 25.0% of genomes.

In the eight plateau isolates, all four vancomycin-like and all defensin-like ARGs were present. Among the plateau isolates, A37 and A44 carried the highest ARG counts (*n* = 9 and *n* = 8, respectively). Notably, in A44, *tet(M)* localized to both the ICE and the T4SS, while *lnuP* was located on the IME.

### Characterization of environmental stress tolerance in *C. perfringens*

**Fig 5:**
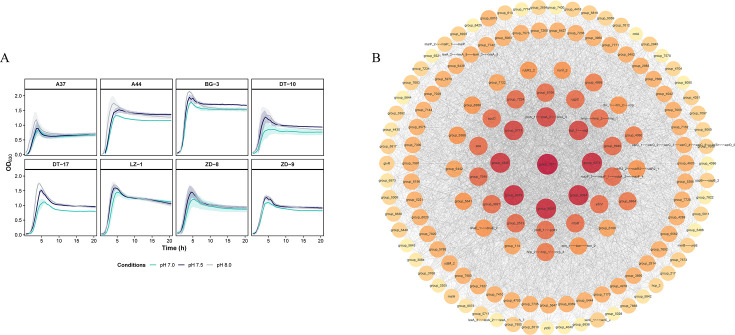
(**A**) This figure illustrates the growth curves of a *Clostridium perfringens* strain cultured under three different pH conditions. Growth was monitored by measuring the OD_630_ over time in cultures maintained at pH 7.0, 7.5, and 8.0. Curves represent the mean across three biological replicates for each condition, and the shaded areas indicate the standard deviation. The *x*-axis represents the incubation time, and the *y*-axis shows the corresponding OD_630_ values, allowing for a direct comparison of pH-dependent growth rates. Growth data were analyzed using a two-tailed paired *t*-test, with statistical significance defined as *P* < 0.05. (**B**) The gene association network reveals the distinct gene interaction patterns between strains stratified by tolerance and sensitivity to different pH levels (tolerant, *n* = 3; nontolerant, *n* = 5). In this network, node size and color saturation represent gene connectivity (degree), where larger, darker nodes indicate higher connectivity. Gray edges denote correlations between genes. The network topology highlights core genes as central hubs, suggesting synergistic metabolic interactions and ecological niche differentiation among strains across pH conditions, thus providing insights into their environmental adaptation mechanisms. Detailed information on the hub genes and their corresponding degree values is provided in Table S4.

We evaluated the acid tolerance of eight *C. perfringens* strains by incubating cultures at pH 7.0, 7.5, and 8.0 and monitoring growth kinetics. Strains grown at pH 7.0 and 7.5 exhibited significantly reduced specific growth rates during the exponential phase and modestly lower maximum cell densities compared to pH 8.0. These results indicate that less favorable pH conditions (near-neutral, pH 7.0–7.5) inhibit *C. perfringens* proliferation and reduce stationary-phase biomass (Fig. 5A). Data are presented as mean ± standard deviation (SD) to reflect biological variability.

We further applied two-tailed paired *t*-tests to statistically evaluate strain-specific differences in environmental adaptability. Comparative analysis revealed that strains A44, ZD-9, and DT-10 maintained similar final cell densities at pH 7.5 despite initially reduced growth rates (*P* ≥ 0.05). In contrast, all isolates displayed a significant decline in stationary-phase biomass under pH 7.0 (*P* < 0.05). These findings highlight the heightened sensitivity of *C. perfringens* to acidic conditions and underscore the inhibitory effects of low pH on its growth and metabolism. This provides a theoretical foundation for developing targeted prevention and control strategies against this pathogen.

**Fig 6:**
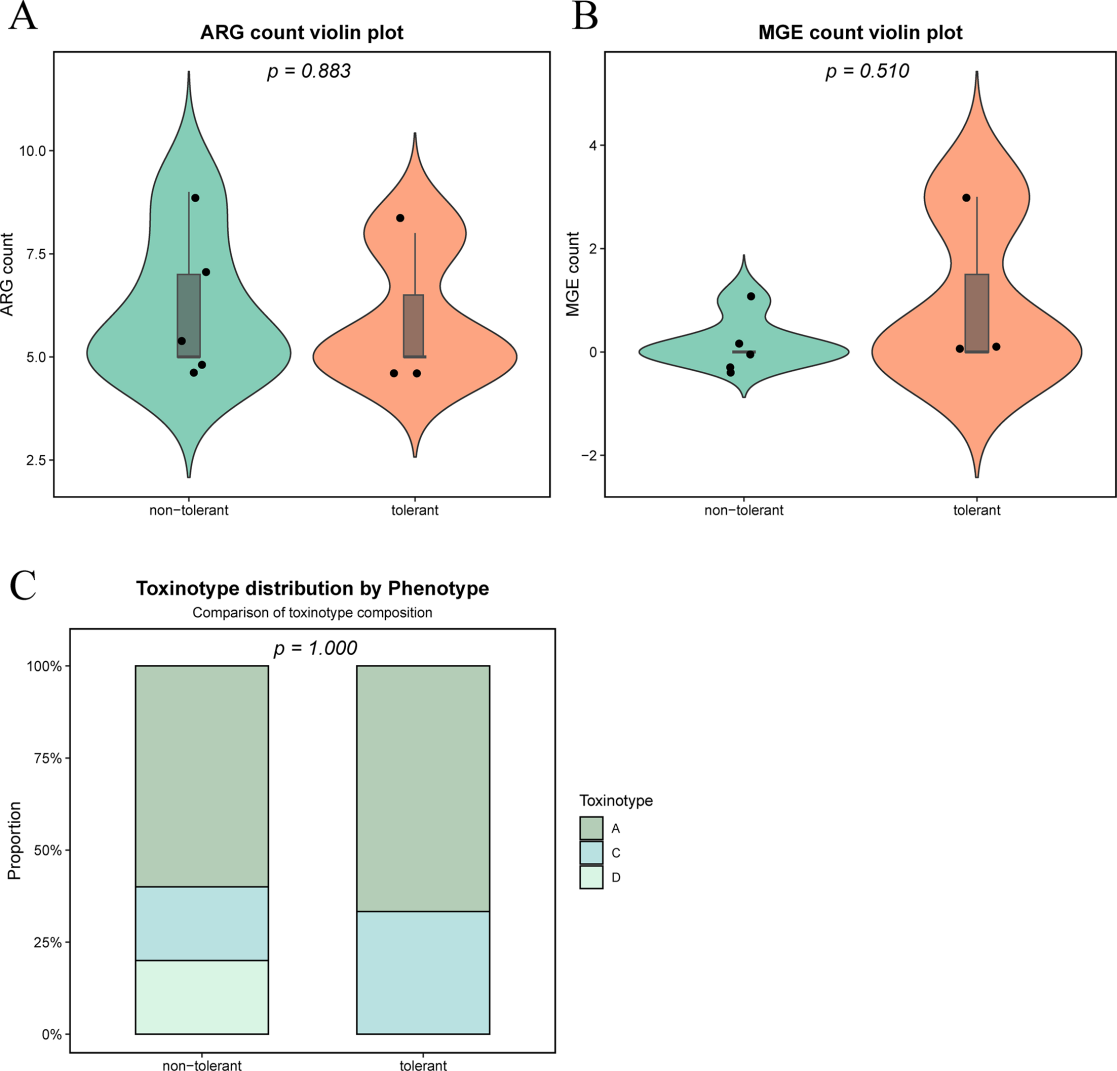
Statistical comparison of genomic features between tolerant and nontolerant *Clostridium perfringens* isolates. (**A and B**) Violin plots displaying the distribution of ARG counts (**A**) and MGE counts (**B**). The width of the violin represents the data density, while the internal box plots indicate the median and interquartile ranges. Black dots represent individual strains. Differences in mean counts between groups were analyzed using unpaired *t*-tests. (**C**) Stacked bar chart showing the proportional distribution of toxinotypes across phenotype groups. The association between toxinotype composition and tolerance phenotype was evaluated using Fisher’s exact test. *P*-values are indicated above each panel. Detailed phenotypic and genotypic data of the plateau strains are provided in Table S3.

To investigate whether broad genomic signatures correlated with the observed environmental tolerance, we compared the toxinotypes, ARG burdens, and MGE counts between the tolerant (*n* = 3) and nontolerant (*n* = 5) groups (Fig. 6). Detailed strain-level genomic data are provided in Table S3. Toxinotypes were distributed across both phenotypic groups; for example, toxinotype A and C strains appeared in both tolerant and nontolerant categories (Fig. 6C). Similarly, while the tolerant strain A44 harbored a high number of ARGs (*n* = 8) and MGEs (*n* = 3), other tolerant isolates (ZD_9 and DT_10) carried fewer resistance determinants (*n* = 5) and lacked identified MGEs, a profile resembling those of several nontolerant strains (e.g., ZD_8 and BG_3). The absence of significant statistical associations between these broad genomic metrics and the tolerance phenotype (supported by *P* > 0.05 in Fig. 6A and B) suggested that environmental adaptation might be driven by specific functional gene combinations rather than total gene counts or toxinotypes.

### Construction of the gene co-occurrence network and node centrality analysis of isolates from the Tibetan Plateau

Based on the classification into tolerant and nontolerant groups across varying pH conditions, gene profiles of eight isolates were obtained from the pan-genomic presence-absence matrix, resulting in 407 candidate genes based on a significance threshold of *P* = 0.5. Subsequently, 3,390 gene-gene associations (threshold: correlation coefficient > 0.5) were identified, involving 146 genes. To systematically elucidate potential gene-gene interactions and identify key hub genes, an association network was constructed in Cytoscape v3.10.3. Degree centrality was employed to quantify each node’s number of direct connections, where a higher degree suggests a more central regulatory role within the network. To systematically resolve the hierarchical organization of the association network and identify key network elements, nodes were stratified by degree (degree centrality). Nodes with degree >80 were defined as core hubs, those with degree between 70 and 80 (inclusive) were defined as secondary hubs, and nodes with degree <70 were designated as low-connectivity nodes. Using these criteria, we selected 23 nodes with degree >70 as the network’s core functional components, including a single highly connected core node, group_7677. We then performed topological and functional annotation analyses on these core components and their immediate neighbors to identify candidate hub genes and infer their potential roles in the stress response. Specifically, the topological arrangement reveals how these genetic elements cluster together. A high “degree centrality” (number of connections) indicates a “hub” gene that acts as a keystone in the genetic network, potentially bridging different functional modules.

Results indicated that the core node, group_7677 (a member of the IS607/IS200IS605 transposase family, *ISCbt3/ISCpe4*), directly connects to 97 other genes, demonstrating pronounced hub characteristics (Fig. 5B). Among the remaining eight core-hub genes (excluding the primary core node group_7677), those with clear annotations were predominantly involved in nucleic acid synthesis and information transfer (e.g., *sigL*, *sigA*, and *polA*). Among the 14 genes designated as secondary hubs were *mazF* toxin-antitoxin genes (*mazF_1, mazF_2, mazF_4, and ndoA_2*), hmp oxidoreductases (*hmp_1 and hmp_2*), metabolism-related genes (*ade and mtaD*), and polyamine/phosphoglycerol transporters (*potB_1, potH, spuD, and ugpC*), suggesting that these elements collaboratively form a core regulatory network supporting *C. perfringens* survival and pathogenicity under stress. In contrast, lower-degree genes showed no significant association with phenotypic tolerance differences and may therefore represent noncentral functional components.

In conclusion, degree centrality analysis identified multiple putative hub genes that may serve as a useful starting point for future investigations into the molecular mechanisms underpinning *C. perfringens* survival, antimicrobial resistance, and virulence regulation.

## DISCUSSION

MLST is commonly used to classify the population diversity of *C. perfringens* in animals, environmental samples, and humans and to compare their genetic relationships. In this study, all genomes were typed using fastMLST. Among the eight *C. perfringens* genomes collected from the Qinghai-Tibet Plateau, three carried novel alleles and five were assigned to new STs. In accordance with the findings of preceding studies, *C. perfringens* exhibits substantial genetic variability in MLST typing (58–61). Based on MLST typing of all genomes, novel alleles represented 25.5% (35/137) of all identified results. And the updated ST scheme yielded 55 distinct STs, further confirming the prevailing view of extensive genetic diversity within this bacterial population.

The excessive use of antimicrobials in livestock, both for controlling *C. perfringens*-induced necrotizing enteritis and as growth promoters, has driven the emergence of AMR strains (62, 63). Resistance in gut microbes, particularly zoonotic pathogens, is now recognized as a major public health concern (64). Our genomic analysis revealed 16 resistance genes across eight antibiotic classes, with vancomycin- and defensin-like determinants present in over 96.0% of isolates. Notably, vancomycin, considered a “last line of defense” against gram-positive infections, showed unexpectedly high resistance prevalence, suggesting strong selective pressure from glycopeptide usage (65). Although glycopeptide resistance in *C. perfringens* has been reported in livestock elsewhere, its high frequency in plateau isolates is striking given the region’s distinctive veterinary antimicrobial practices. Furthermore, the detection of three ARGs associated with MGEs or type IV secretion systems from plateau isolates highlights the potential for horizontal transfer, a finding consistent with those of previous research indicating the potential for horizontal gene transfer among plateau bacteria (32), underscoring the need for targeted surveillance and stricter antimicrobial stewardship, even in relatively remote ecosystems.

Phenotypic assays revealed that a subset of plateau isolates maintained growth at pH 7.5 under anaerobic incubation, suggesting adaptation to fluctuating gastrointestinal or environmental pH. To decipher the genetic architecture underlying this adaptation, we constructed a gene co-occurrence network. Comparative pan-genomic analysis between tolerant and nontolerant isolates identified a dense gene-gene association network (146 nodes; 3,390 edges), with central hubs including *IS* family transposases (*ISCbt3/ISCpe4*), sigma factors (*sigL* and *sigA*), and DNA polymerase I (*polA*).

IS family transposases (*ISCbt3/ISCpe4*) emerged as core hubs, facilitating adaptive genome plasticity via noncanonical excision-integration under high-altitude stresses like hypoxia, UV exposure, and diurnal temperature shifts (27, 28, 66–72). These elements associate with toxin and antibiotic-resistance loci (e.g., *cpe, netB, and tet*) to facilitate their mobilization (73, 74), while also altering genome structure and modulating transcription by impacting promoters or regulatory sequences (75, 76).

Concurrently, the centralization of sigma factors (*sigA and sigL*) and DNA polymerase I (*polA*) as network hubs suggests a coordinated response involving transcriptional reprogramming and genome maintenance. The housekeeping factor *sigA* and the stress-responsive *sigL* enable global transcriptional adaptation (77–82), with *sigL* specifically linking nutrient utilization and metabolic flexibility to virulence and biofilm formation under constraints like oxygen limitation and resource scarcity (83–85). Furthermore, the hub position of *polA*, essential for replication and excision repair (86–89), reflects a critical need to maintain genome integrity against plateau-associated DNA damage from elevated UV radiation and oxidative stress (88, 89). Collectively, these hubs form an adaptive module integrating genome plasticity, transcriptional control, and maintenance under high-altitude selective pressures.

Although bioinformatics tools can reveal gene co-occurrence networks among different *C. perfringens* strains, experimental validation is still needed to determine their precise functions in this pathogen. Key questions remain regarding how these genes influence virulence gene expression, dissemination, or genomic rearrangement and how they contribute to the strain’s ability to adapt to diverse environmental conditions, all of which represent important directions for future research. Future studies should integrate large-scale sample collection, *in vitro* functional assays, and the construction of gene knockout mutants to further elucidate the mechanisms underlying antimicrobial resistance, virulence evolution, and environmental tolerance in this pathogen.

## Data Availability

The sequence data for the strains investigated in this study have been submitted to the NCBI database under the BioProject accession number PRJNA1346701. Information on other public sequences used in this study is detailed in Table S1. All software versions, database releases, and key parameters are summarized in Table S2. Detailed strain-level phenotypic and genotypic data for the plateau isolates are provided in Table S3. Detailed information on the hub genes and their corresponding degree values is provided in Table S4.

## SUPPLEMENTAL MATERIAL

Supplemental tables msphere.00792-25-s0001.xlsx Tables S1 to S4.

ASM does not own the copyrights to Supplemental Material that may be linked to, or accessed through, an article. The authors have granted ASM a non-exclusive, world-wide license to publish the Supplemental Material files. Please contact the corresponding author directly for reuse.
